# Expression of Ethanol-Induced Behavioral Sensitization Is Associated with Alteration of Chromatin Remodeling in Mice

**DOI:** 10.1371/journal.pone.0047527

**Published:** 2012-10-22

**Authors:** Béatrice Botia, Rémi Legastelois, Stéphanie Alaux-Cantin, Mickaël Naassila

**Affiliations:** Université de Picardie Jules Verne, Unité de Formation et de Recherche de Pharmacie, Research Group on Alcohol and Pharmacodependences, Institut National de la Santé et de la Recherche Médicale (ERI 24), Amiens, France; Mayo Clinic College of Medicine, United States of America

## Abstract

**Background:**

Ethanol-induced behavioral sensitization (EIBS) is proposed to play a role in early and recurring steps of addiction. EIBS does not occur uniformly in all animals even from the same inbred strain. Since recent data demonstrate that epigenetic mechanisms are likely to be involved in the development and the persistence of ethanol-related behaviors, we explored the involvement of epigenetic mechanisms in ethanol response after EIBS development.

**Methodology:**

DBA/2J mice were i.p. injected with saline or ethanol (2 g/kg) once a day for 10 consecutive days. At day 17, ethanol-treated mice were split in resistant and sensitized groups. Brains were then removed 30 min after a saline or 2 g/kg ethanol challenge to assess *i)* gene expression using PCR array targeting 84 epigenetic-related genes and *ii)* histone deacetylases (HDAC), histone acetylases (HAT) and DNA methyltransferases (DNMT) activities as well as H4K12 acetylation.

**Principal Findings:**

Acute ethanol administration decreased *dnmt1, esco2* and *rps6ka5* genes expression. These genes were similarly altered in sensitized but not in resistant mice after an ethanol challenge, suggesting that resistant mice were tolerant to the transcriptional outcomes of an ethanol challenge. Whereas global HAT or DNMT activity was not affected, global HDAC activity was reduced after an acute ethanol injection. HDAC inhibition occurred in all ethanol-treated mice but with a lesser extent in sensitized animals. As a consequence, H4 acetylation was specifically potentiated in the core of the Nac proportionally to the striatal HDAC activity decrease.

**Conclusions/Significance:**

The present study highlights that the contrasted behavioral response to an ethanol challenge between resistant and sensitized mice may be mediated by epigenetic mechanisms occurring specifically in the striatum. Here we show that vulnerability to ethanol dependence and relapse could be, at least in part, due to individual variability in acute ethanol-induced epigenetic response.

## Introduction

Among the different theories that could explain addiction, the incentive salience sensitization theory states that repeated exposure to drugs of abuse causes hypersensitivity to drugs and drugs-associated stimuli of the neuronal circuits mediating incentive salience, an important way in which motivational stimuli influence behavior [1]. Ethanol-induced behavioral sensitization (EIBS) is defined as a progressive enhancement of motor stimulant effect following repeated ethanol administrations [2], [3].

Behavioral sensitization displays two phases that differ at the anatomical level. The induction phase is described as a progressive enhancement of the locomotor activity induced by ethanol and involves the ventral tegmental area. The expression phase corresponds to the enduring behavioral hypersensitivity to ethanol after the cessation of treatment and involves the ventral striatum (nucleus accumbens, Nac) [4], [5]. The induction and expression phases of EIBS also differ at the pharmacological level. Indeed, naloxone, disulfiram and D3 antagonists decrease the magnitude of EIBS induction but have no effect on EIBS expression [6]–[8]. Conversely, CRF1 antagonism has no effect on induction but blocks EIBS expression [9]. The neuroadaptations underlying sensitization expression require a withdrawal period for their full development and are proposed as a relevant process in the recurring stages of alcohol addiction. In particular, neuroadaptations occurring after behavioral sensitization and triggered by an ethanol challenge also play a role in relapse to drug seeking [1]–[3].

Whereas behavioral sensitization is a robust phenomenon observed in several species, critical individual differences are described in the development and magnitude of expression of EIBS between individuals [10]–[14]. Indeed, some mice exhibited clear signs of sensitization (‘sensitized mice’) and others similarly treated did not display progressive enhancement of locomotor activity during EIBS procedure (non-sensitized or resistant mice). As a consequence, these contrasted responses provide an opportunity to discriminate the global pharmacological effects of ethanol from effects specifically associated with sensitization processes. Therefore, focusing our research on the expression phase of sensitization allows us to only consider the sensitized-specific adaptations putatively involved in relapse to drug-seeking. To date, only few studies investigated differential neurobiological changes induced by repeated ethanol administrations in sensitized and resistant mice and attention has been given to the neurotransmitter systems. Based on their locomotor activity score (last day/first day), the ethanol-treated mice were classified as sensitized and non-sensitized. Resistant mice exhibit significant higher NMDA [12], [13] and lower D2 [14] receptors binding in specific brain areas, such as Nac core, prefrontal cortex (PFC) or caudate-putamen, when compared to sensitized mice. Understanding the neurochemical machinery that may underlie these differences is a priority for in-depth investigation of EIBS reversal.

Increasing attention has been given to the role of epigenetic mechanisms in modulating gene expression, leading to various behavioral and physiological ethanol responses. Indeed, epigenetics can explain long-lasting changes in gene expression and recent studies also indicated that ethanol alters the activity of the different enzymes involved in chromatin remodeling. Particularly, acetylation of histone proteins appears as a crucial mechanism in the development of alcohol addiction. Acute ethanol treatment decreases histone deacetylases (HDAC) activity and increases acetylation of histone H3 and H4 in rat amygdaloid brain regions. In contrast, repeated ethanol administration alters neither HDAC activity nor histone acetylation. However, withdrawal after chronic ethanol treatment increases HDAC activity and decreases histone acetylation [15], [16]. Moreover, adolescent rats submitted to ethanol intermittent administrations exhibit an increase in H3 and H4 acetylation, in H3 dimethylation and in histone acetyltransferases (HAT) activity in the PFC [17]. These studies strongly support an alteration of epigenetic mechanisms after both acute and chronic ethanol treatment. Whereas recent data suggest a role of HDACs in EIBS acquisition [18], to date, epigenetics involvement in response to an ethanol challenge after sensitization has not been investigated.

In this context, we carried out an exploratory analysis to identify some regulations of striatal expression of epigenetic-related genes in resistant and sensitized mice during EIBS expression phase. In a separate experiment, we investigated the outcome of prior behavioral sensitization on ethanol challenge response regarding HDAC, HAT and DNMT activities and histone H4 acetylation in brain structures involved in reinstatement (striatum, PFC, amygdala and hippocampus; [19]). We postulated that both approaches would extend knowledge on the involvement of epigenetic mechanisms not only in the persistence of ethanol-related behaviors such as EIBS expression but also on vulnerability to ethanol addiction.

## Results

### DBA/2J Mice Display Individual Differences in Behavioral Sensitization to Ethanol

**Figure 1:**
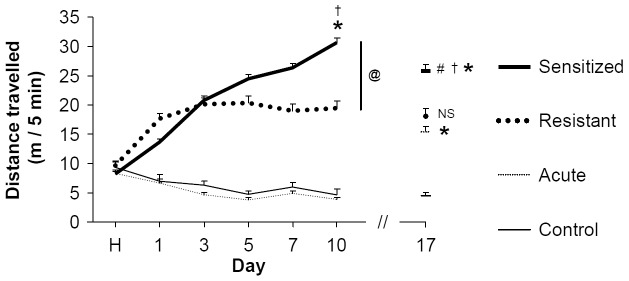
Individual variability in EIBS development. On the first day of the experiment (habituation day, H), all mice were injected with saline solution (n = 60). Then, from days 1 to 10, mice received 10 consecutive once-daily i.p. injections of saline (n = 20) or 2 g/kg ethanol (n = 40) solution immediately followed by a 5 min-long locomotor activity recording session. Some mice exhibited EIBS (sensitized mice, n = 29) whereas others failed to sensitize (resistant mice, n = 11). At day 17 (challenge day) mice received a single injection of saline (control group) or 2 g/kg ethanol (acute, resistant and sensitized groups). * indicates an increase *vs* day 1. † indicates a significant difference between resistant and sensitized mice at the same day. # indicates significant differences between sensitized and acute mice at day 17. @ indicates significant differences between resistant and sensitized groups throughout the induction phase.

A 1-way ANOVA failed to detect a difference in locomotor activity at day H between control, resistant and sensitized mice (F[3,134] = 1.435; *P* = 0.235) **(** **Figure 1** **)**. These findings suggest that resistant mice failure to manifest sensitization is not due to impaired locomotor or exploratory abilities.

Since no difference was detected between acute and control groups during the induction phase (day 1–day 10), data from both groups were pooled to performed the first 2-way RM-ANOVA focused on EIBS induction. Statistical analysis highlighted an effect of group (F[2,135] = 217.170; *P<*0.001), an effect of day (F[4,535] = 41.593; *P<*0.001) and a significant interaction (F[8,535] = 65.527; *P<*0.001) **(** **Figure 1** **)**. *Post-hoc* tests revealed a significant increase in locomotor activity between day 1 and day 10 for the sensitized (*P<*0.001) but not for the resistant groups (*P = *0.265) and showed a slight habituation in the control group (*P*<0.01).

At day 17, mice received one injection of saline or 2 g/kg ethanol **(** **Figure 1** **)**. A 2-way RM-ANOVA focused on days 1, 10 and 17 revealed an effect of group (F[3,134] = 90.467; *P<*0.001), an effect of day (F[2,268] = 21.060; *P<*0.001) and a significant interaction (F[6,268] = 51.943; *P<*0.001). *Post-hoc* tests indicated, as expected, that mice injected with saline solution during the induction phase exhibited a significant increase in locomotor activity at day 17 in response to ethanol (acute group; *P<*0.001 *vs* D1). Sensitized mice were still sensitized on day 17 after ethanol challenge (*P<*0.001 *vs* D1; *P<*0.001 *vs* acute mice at D17). Resistant mice were still unsensitized at day 17 after ethanol challenge (*P* = 0.903 *vs* D1; *P* = 0.332 *vs* acute mice at D17; *P<*0.001 *vs* sensitized mice at D17 **(** **Figure 1** **)**. A 1-way ANOVA detected no difference between groups neither for BECs (F[2], [24] = 1.233; *P = *0.309; **Figure S2a**), nor for mice body weight at day 17 (F[2], [35] = 0.654; *P* = 0.526; **Figure S2b**). Thus, it is unlikely that the behavioral differences detected between resistant and sensitized mice may be related to changes in ethanol metabolism or food intake.

### Expression of EIBS is Associated with Tolerance to Ethanol Challenge Transcriptional Effects on Epigenetic-related Genes

**Figure 2:**
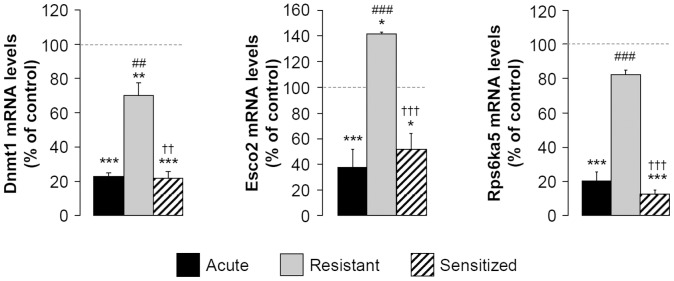
Expression of EIBS is associated with tolerance to the transcriptional effects of an ethanol challenge on epigenetic-related genes. During 10 days, mice received daily i.p. injections of saline or ethanol (2 g/kg) solution. At day 17, 30 min after saline or ethanol challenge (2 g/kg), striata were isolated to perform real-time PCR (n = 4 per group). Means (± SEM) of regulated genes expression are expressed as percentages of control *P<0.05, **P<0.01, ***P<0.001 *vs* control; ^##^ *P<*0.01, ^###^ *P<*0.001 *vs* acute; ^††^ *P<*0.01, ^†††^ *P<*0.001 *vs* resistant groups.

A single injection of 2 g/kg ethanol (acute group) altered the expression of 21 genes involved in histone modification and DNA methylation. Among these genes, 5 were up- and 16 were down-regulated **(Table S1)**. Nineteen of these genes were similarly regulated after an ethanol challenge at day 17 in sensitized mice **(Table S1)**. On the contrary, 12 of these transcripts were unaltered in resistant mice after an ethanol challenge at day 17 **(Table S1)**. Even when genes were regulated in the same direction between acute and resistant mice, our results emphasized significant differences in the regulation levels. Including all these transcripts, 19 were significantly different between acute/sensitized and resistant mice (**Table 1**). These 19 genes were identically regulated between the acute and sensitized groups whereas these regulations were significantly less important in the resistant group (**Table 1**). Thus, these data suggest that resistant mice were mostly tolerant to some transcriptional effects of an ethanol challenge. We only represented genes whose regulation passed the Bonferroni’s correction in **Figure 2**. The expression of *dnmt1*, *esco2* and *rps6ka5* dramatically decreased in acute groups (−77%, −62%, −80% under control, respectively). Similar regulations were observed in sensitized but not in resistant mice that appear tolerant to the transcriptional outcomes of an ethanol challenge (+204%, +274%, +310% over acute, respectively). Moreover, *esco2* was strongly reduced in acute and sensitized groups and increased in resistant mice striata suggesting that resistance to EIBS was associated not only with a reduced ethanol transcriptional effect but also with opposite effects **(** **Figure 2** **)**.

**Table 1:** Ethanol challenge in resistant and sensitized mice is associated with striatal epigenetic-related genes regulation.

	Acute	Sensitized	Resistant	Resistant	
Gene	*vs* control			*vs* acute	*vs* sensitized
**Dnmt1**	↓↓↓	↓↓↓	↓↓	↑↑	↑↑
**Esco2**	↓↓	↓	↑	↑↑↑	↑↑↑
Hdac11	↓↓↓	↓↓↓	=	↑↑	↑↑↑
Prmt5	↓	↓↓	=	↑↑	↑↑↑
Prmt6	↓↓↓	↓↓↓	↓	=	↑
Prmt7	↓↓↓	↓↓↓	=	↑↑↑	↑↑
**Rps6ka5**	↓↓↓	↓↓↓	↓	↑↑↑	↑↑↑
Setd6	↓↓↓	↓↓	=	↑↑	↑↑
Smyd3	↓	↓	=	↑↑	↑↑
Usp21	↓↓	↓↓	=	↑	↑
Hdac10	=	=	=	=	↑
Myst1	=	=	=	↑↑	↑↑
Myst2	=	=	=	↑	↑
Noca6	=	=	↑	=	↑
Rnf2	=	=	=	↑	↑
Setd1a	=	=	=	↑↑	↑↑
Setd3	=	=	=	=	↑
Setd4	=	=	=	=	↑
Usp16	=	=	↑↑	=	↑

During 10 days, mice received daily i.p. injections of saline or ethanol (2 g/kg) solution. At day 17, 30 min after saline or ethanol challenge (2 g/kg), striata were isolated to perform real-time PCR (n = 4 per group). Only genes whose expression is significantly different between resistant and sensitized mice are represented and expressed as percentages of control (mean ± SEM). ↓ and ↑ represent a significant decrease or increase in gene expression, respectively. ^↓,↑^ *P<*0.05, ^↓↓,↑↑^ *P<*0.01, ^↓↓↓,↑↑↑^ *P<*0.001, = *P>*0.05. Regulations that pass the Bonferroni correction are indicated in bold.

These regulations appeared gene specific since some genes were similarly regulated between all groups of mice, showing that resistant mice could also exhibit transcriptional effect of an ethanol challenge (atf2, kdm4a and setdb1; **Table S1**).

### Expression of EIBS is Associated with Changes in Global HDAC Activity in Striatum

**Figure 3:**
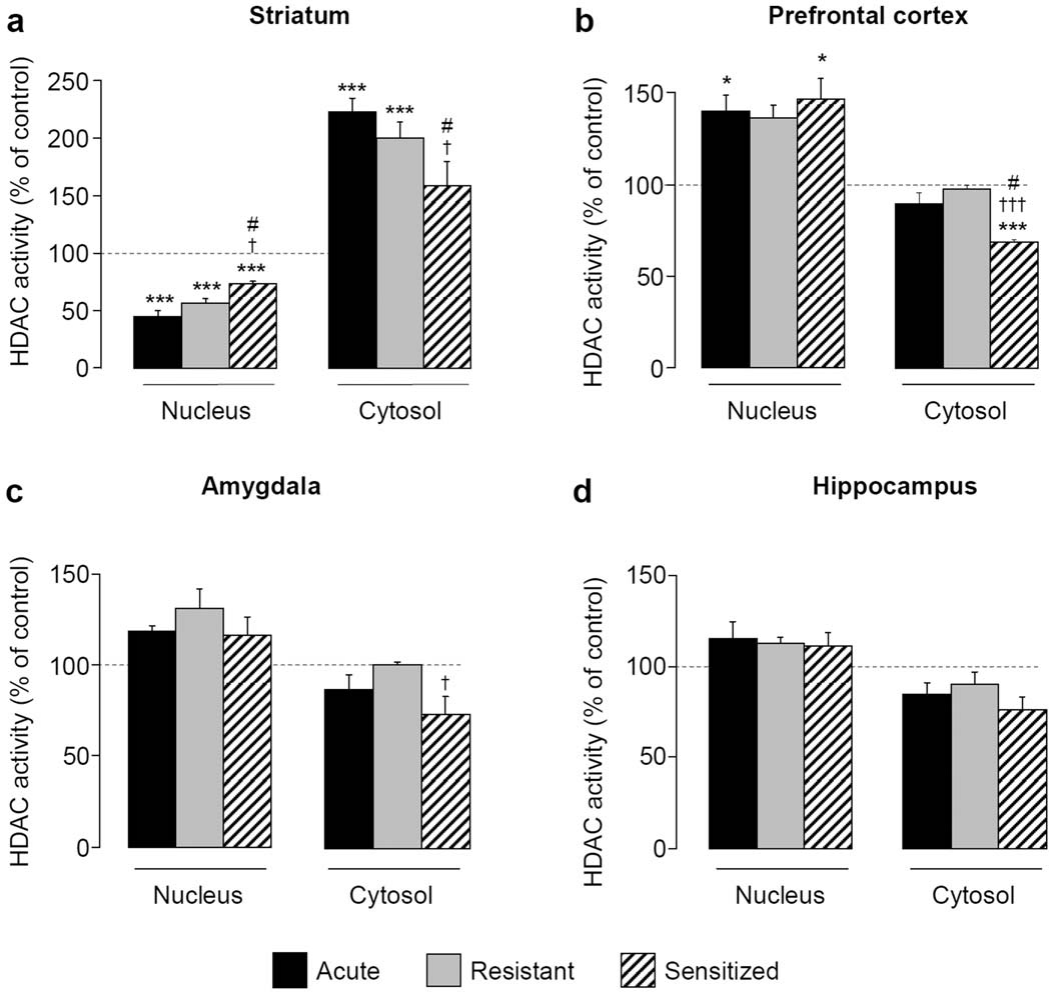
Expression of EIBS is associated with changes in global HDAC activity in striatum. During 10 days, mice received daily i.p. injections of saline or ethanol (2 g/kg) solution. HDAC activity was measured in cytosolic and nuclear fractions of striatum (**a**), prefrontal cortex (**b**), amygdala (**c**) and hippocampus (**d**) 30 min after saline or ethanol (2 g/kg) challenge at day 17 (n = 4–5 per group). Means (± SEM) of HDAC activity are expressed as percentages of control *P<0.05, ***P<0.001 *vs* control; ^#^ *P<*0.05 *vs* acute; ^†^ *P<*0.05, ^†††^ *P<*0.001 *vs* resistant groups.

In the striatum, a 1-way ANOVA highlighted an effect of group in the nuclear (F[3], [12] = 17.783, *P<*0.001) and cytosolic (F[3], [15] = 13.696, *P<*0.001) fractions **(** **Figure 3a** **)**. In the nuclear fraction, *post-hoc* tests showed that HDAC activity was remarkably reduced in acute, resistant and sensitized groups whereas in the cytosolic fraction only acute and resistant mice exhibited a robust increase in HDAC activity. Whatever the fraction these alterations were moderated in the sensitized groups, *i.e*. HDAC activity was higher in the nuclear fraction (78% and 34% over acute and resistant mice, respectively) and lesser in the cytosolic fraction (27% and 20% under acute and resistant mice, respectively, P<0.05) **(** **Figure 3a** **)**. These results suggested that sensitized mice were at least partially tolerant to the ethanol challenge in the nuclear and in the cytosolic fractions, respectively.

In the PFC, statistical analysis highlighted an effect of group in both nuclear (F[3], [16] = 5.049, *P<*0.05) and cytosolic fractions (F[3], [15] = 9.777, *P<*0.001). *Post-hoc* tests showed an increase in HDAC activity in the nuclear fraction of acute (P<0.05) and sensitized (P<0.05) groups and a reduction in the cytosolic fraction of sensitized mice (23% and 29% under acute and resistant mice, *P<*0.05 and *P<*0.001, respectively) **(** **Figure 3b** **)**.

In the amygdala, data analysis showed an effect of group in the cytosol (F[3], [16] = 3.558, *P<*0.05) but not in the nuclear fraction (F[3], [16] = 2.725, *P = *0.079). *Post-hoc* tests revealed a reduced HDAC activity in the cytosolic fraction of sensitized compared to resistant mice (27% under resistant, *P<*0.05; **Figure 3c**).

In the hippocampus, a 1-way ANOVA detected no effect of group on HDAC activity neither in the nuclear (F[3], [15] = 3.282, *P = *0.050) nor in the cytosolic fractions (F[3], [16] = 1.781, *P = *0.191) **(** **Figure 3d** **)**.

### Expression of EIBS is not Associated with Changes in Global HAT or DNMT Activities

**Figure 4:**
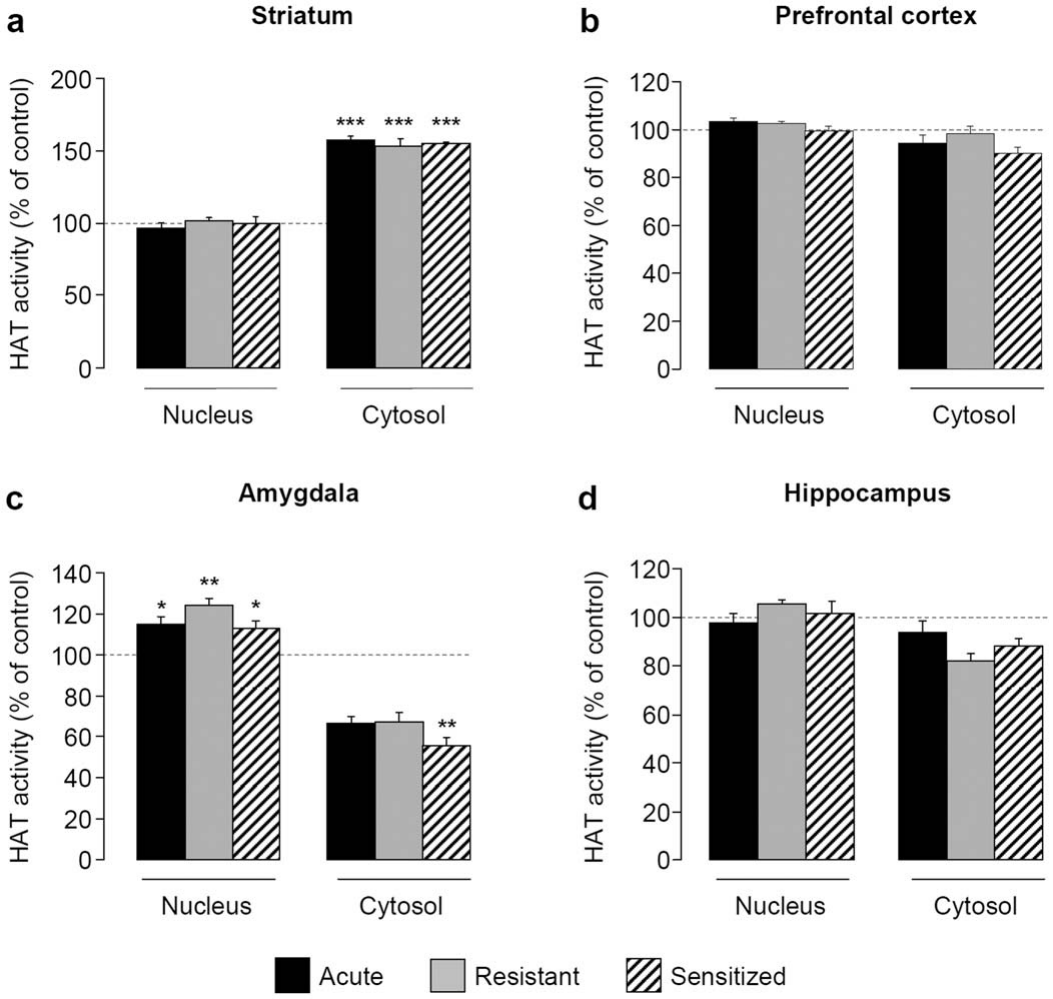
Expression of EIBS is not due to changes in global HAT activity. During 10 days, mice received daily i.p. injections of saline or ethanol (2 g/kg) solution. HAT activity was measured in cytosolic and nuclear fractions of striatum (**a**), prefrontal cortex (**b**), amygdala (**c**) and hippocampus (**d**) 30 min after saline or ethanol challenge (2 g/kg) at day 17 (n = 4–5 per group). Means (± SEM) of HDAC activity are expressed as percentages of control *P<0.05, **P<0.01, ***P<0.001 *vs* control group.

In the striatum, a 1-way ANOVA failed to detect an effect of group on HAT activity in the nuclear fraction (F[3], [16] = 0.235, *P = *0.870) but showed an effect of group in the cytosolic fraction (F[3], [16] = 26.296, *P<*0.001). Indeed, *post-hoc* tests demonstrated that HAT activity increased in the striatal cytosolic fraction of acute, resistant and sensitized groups (about 50% over control, *P<*0.001) **(** **Figure 4a** **)**.

In the PFC, statistical analysis failed to detect an effect of group on HAT activity whatever the fraction (F[3], [16] = 10.407, *P = *0.444 and F[3], [16] = 2.270, *P = *0.120 in the nuclear and cytosolic fractions, respectively) **(** **Figure 4b** **)**.

In the amygdala, data analysis showed an effect of group on HAT activity in both the nuclear (F[3], [16] = 10.407, *P<*0.001) and in the cytosolic fractions (F[3], [16] = 5.106, *P<*0.05). *Post-hoc* tests demonstrated that HAT activity slightly increased in acute (*P<*0.01), resistant (*P<*0.001) and sensitized (*P<*0.01) groups compared to controls in the nuclear fraction and a dramatic reduction in the cytosolic fraction of sensitized mice (54% under control, *P<*0.05) **(** **Figure 4c** **)**.

In the hippocampus, ethanol treatment had no effect on HAT activity whatever the fraction studied (F[3], [16] = 10.407, *P = *0.512 and F[3], [16] = 2.270, *P = *0.076 in the nuclear and cytosolic fractions, respectively) **(** **Figure 4d** **)**.

**Figure 5:**
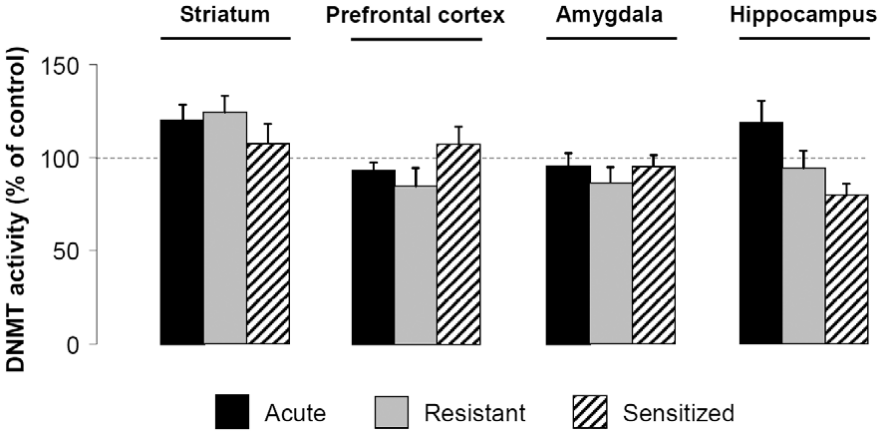
Expression of EIBS is not due to changes in global DNMT activity. During 10 days, mice received daily i.p. injections of saline or ethanol (2 g/kg) solution. DNMT activity was measured in nuclear fractions of striatum, prefrontal cortex, amygdala and hippocampus 30 min after saline or ethanol (2 g/kg) challenge at day 17 (n = 5 per group). Means (± SEM) of DNMT activity are expressed as percentages of control.

A 1-way ANOVA detected no effect of group on DNMT activity in the striatum (F[3], [16] = 1.616, *P* = 0.225), in the PFC (F[3], [16] = 1.561, *P* = 0.238), in the amygdala (F[3], [16] = 0.561, *P* = 0.648) or in the hippocampus (F[3], [16] = 1.726, *P* = 0.202) **(** **Figure 5** **)**.

### Expression of EIBS is Associated with Changes in H4K12 Acetylation in the Core of the Nac

**Figure 6:**
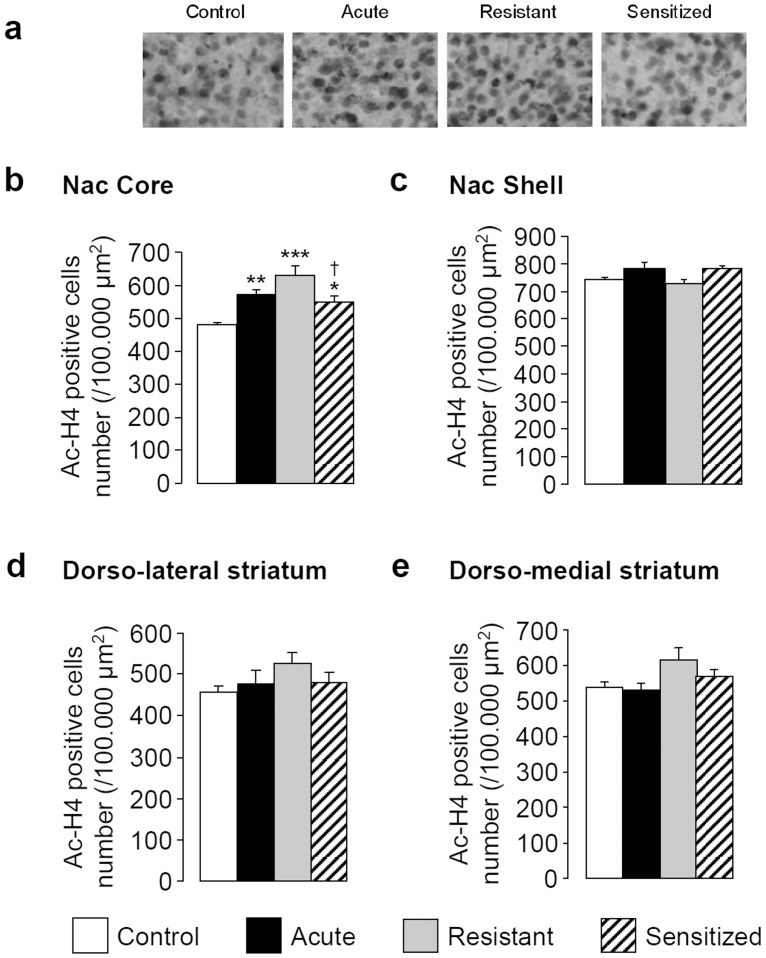
Expression of EIBS is associated with changes in H4K12 acetylation in the core of the Nac. During 10 days, mice received daily i.p. injections of saline or ethanol (2 g/kg) solution. At day 17, mice were challenged with ethanol (2 g/kg) or saline solution and transcardially perfused 30 min later to perform immunohistochemistry (n = 4 per group). (**a**) Photomicrographs illustrating the immunolabelling for ac-H4K12 in the core of the Nac. The number of ac-H4K12 positive cells are represented as mean values (± SEM) in the core (**b**) and in the shell (**c**) of the Nac or in the dorso-lateral (**d**) and dorso-medial (**e**) striatum. *P<0.05, **P<0.01, ***P<0.001 *vs* control; ^†^ *P<*0.05 *vs* resistant groups. Nac, nucleus accumbens.

In the core of the Nac **(** **Figure 6a, b** **)**, a 1-way ANOVA showed a main effect of group (F[3], [12] = 14.735, *P<*0.001) and *post-hoc* tests highlighted an increase in the number of positively labelled nuclei for ac-H4 in acute (*P<*0.01), resistant (*P<*0.001) and sensitized (*P<*0.05) compared to control groups. Interestingly, the augmentation in the sensitized group was significantly lower than in the resistant group (13% under resistant; *P<*0.05).

A 1-way ANOVA failed to detect any effect of group on H4 acetylation in the shell of the Nac (F[3], [12] = 2.258, *P = *0.134; **Figure 6c**), in the dorso-lateral (F[3], [12] = 1.205, *P = *0.350; **Figure 6d**) or in the dorso-medial striatum (F[3], [12] = 2.925, *P = *0.077; **Figure 6e**).

Quantification of NeuN positive cells number did not reveal any differences between groups in the core (F[3], [12] = 0.689, *P = *0.576; **Figure S3a, b**) or in the shell of the Nac (F[3], [12] = 0.0462, *P = *0.986; **Figure S3c**) and in the dorso-lateral (F[3], [12] = 0.485, *P = *0.699; **Figure S3d**) or in the dorso-medial striatum (F[3], [12] = 1.428, *P = *0.283; **Figure S3e**).

## Discussion

As previously described in humans and rodents [10], [12]–[14], our results confirmed individual differences in response to ethanol. Indeed, we emphasized that high levels of locomotor stimulation emerged after chronic ethanol treatment in some mice but not in others genetically identical and similarly treated. We proved that differential expression of EIBS is not linked to alteration in ethanol metabolism, motor coordination or exploratory activity. Identification of the mechanisms underlying resistance to EIBS becomes of intense interest since it would provide information about neurobiological factors that generate the heterogeneity in alcohol-responsiveness, potential vulnerability to alcohol addiction and relapse.

Several studies explored the impact of chromatin remodeling in ethanol-related behaviors [16], [17]. For instance, HDAC inhibition counters anxiety associated with ethanol-induced withdrawal [15]. However, regarding EIBS, the role of epigenetic mechanisms with respect to their molecular targets has to be further investigated. In this context, we performed PCR experiments targeting 84 epigenetic-related genes during the expression phase after an ethanol challenge in striatum, because of its crucial role in sensitization expression. We showed that all genes whose expression was statistically different between resistant and sensitized mice were regulated following the same pattern. Indeed, the expression of *dnmt1*, *esco2* and *rps6ka5* was strongly reduced in acute and sensitized mice but less or not in resistant animals. This observation suggests that resistance to EIBS may be closely related to resistance to ethanol-induced epigenetic-related gene expression regulations. However, it is still unknown whether these differences in the sensitivity to ethanol transcriptional effects between resistant and sensitized mice are predisposing factors or responses to EIBS development. It is obvious that the acute ethanol group contains both EIBS resistant and prone mice. As the acute ethanol group does not exhibit intermediate genes expression levels in comparison to the resistant and sensitized groups, we hypothesize that the observed regulations in resistant mice are not pre-existing but develop after chronic ethanol treatment and it would be interesting to further investigate this point.

Dnmt1, the most abundant among the DNA methyltransferase can regulate *de novo* and maintenance DNA methylation processes in cells [20]. DNA methylation is also thought to mediate the long-lasting changes in gene expression that occurs with repeated drug of abuse exposure [21], [22] as demonstrated by the delayed development of cocaine-induced behavioral sensitization induced by DNMT activity inhibition [21]. Esco2 is an acetyltransferase which interacts with various chromatin modifying enzymes, such as histone methyltransferases, demethylases and deacetylases [23]. Rps6ka5 (also called MSK1) is highly expressed in mouse and human brains, especially in the striatum [24] and is involved in H3 phosphorylation [25]. Literature underlines that *rps6ka5* is essential for the development of cocaine sensitization [26]. Our results emphasized a role of these genes in EIBS expression and thus provide new putative targets for EIBS reversal. Interestingly, our results also highlighted that acute ethanol treatment regulated the expression of some epigenetic-related genes like *atf2, hdac2* or *hdac11*. These results are in accordance with previous studies showing that HATs and HDACs are regulated by drugs of abuse. For example, cocaine self-administration is accompanied by an increase in HDAC2 and HDAC11 synthesis [27] and methamphetamine injection modulates ATF2, HDAC1 and HDAC2 protein levels in the Nac [28].

In a separate experiment, we investigated whether resistant or sensitized mice display differential response to the effect of an ethanol challenge on epigenetic-related enzymes activity. It is noteworthy that we measured global enzymatic activities and thus we can not identify which specific enzyme is predominantly involved. Albeit global HAT or DNMT activities were not associated with vulnerability to EIBS, resistant and sensitized mice exhibited significant differences in nuclear and cytosolic striatal HDAC activity. Interestingly, the more HDAC activity decreased in the nuclear fraction, the more it increased in the cytosolic fraction, suggesting a potential translocation of some HDACs in response to ethanol. Indeed, HDACs from class II and IV (HDAC11) shuttle between cytoplasm and nucleus [29]. More investigations are required to explore this hypothesis. It is not surprising that acute ethanol treatment causes both inhibition and potentiation of nuclear HDAC activity in the striatum and PFC, respectively. Indeed, previous studies have shown that ethanol can have different or even opposite effects on chromatin remodeling depending of the considered structure. For instance, in adolescent rats chronically treated with ethanol, H4 acetylation levels decrease of in the frontal cortex, increase in the striatum and remain unchanged in the hippocampus [30]. The decrease of cytosolic HDAC activity in several structures (i.e. striatum, PFC and amygdala) of sensitized animals compared to resistant, suggests the potential involvement of HDAC-related non-epigenetic mechanisms in vulnerability to EIBS. Indeed, HDAC inhibition can also regulate non-transcriptional events, such as improvement of microtubule stability *via* enhanced acetylation [31].

We next hypothesized that changes in HDAC activity observed after ethanol challenge could induce histone H3 and H4 covalent modifications as previously described [15], [32]. We focused our research on histone H4 since several studies highlighted the influence of H4 acetylation on addiction-related behaviors [33], [34]. Moreover, Pandey and colleagues demonstrated similar regulations in H4 and H3 acetylation in the process of alcohol withdrawal [15]. Since differences in HDAC activity between sensitized and resistant mice occurred specifically in the striatum, we assessed H4 acetylation in 4 striatal sub-structures (i.e. core and shell of the Nac and dorso-lateral and dorso-medial striatum). The present study demonstrated that ethanol challenge caused an increase in H4 acetylation specifically in the core of the Nac, with or without prior repeated ethanol treatment. This global increase was inversely proportional to the HDAC activity decrease in the striatum, supporting a functional role of HDAC activity alterations. Our results demonstrating a specific involvement of Nac core in EIBS are in line with previous studies highlighting its specific role in drug of abuse-induced behavioral sensitization [35]–[37]. Moreover, resistance to ethanol sensitization is associated with increased NMDA receptor binding specifically in the Nac core [12]. Our results are also in accordance with previous studies showing that acute ethanol treatment causes a decrease in HDAC activity and an increase in H4 acetylation in rat amygdaloid brain regions [15], [16]. A recent study showed that re-exposure to the same ethanol dose causes tolerance to ethanol-induced epigenetic alterations and that a HDAC inhibitor countered this tolerance [16]. Our results suggested the development of tolerance to the ethanol response of epigenetic-related enzymes in sensitized compared to resistant mice. As a consequence, we supposed that the development of EIBS could be dependent upon tolerance to ethanol-induced epigenetic modifications. Conversely, the lack of tolerance could therefore lead to EIBS resistance. It was not surprising that resistance to EIBS was associated with a tolerance to the transcriptional outcomes of an ethanol challenge and with a lack of tolerance to HDAC activity inhibition and subsequent increase in H4 acetylation. It is obvious that ethanol-induced transcriptional alterations on epigenetic-related genes are not directly linked to the inhibitory effect of ethanol on HDAC activity. It is clear that the goal of the PCR experiments was to explore the transcriptional outcome of ethanol challenge on epigenetic-related genes, rather than the consequences of ethanol-induced HDAC activity modulations on subsequent gene expression alteration through chromatin remodeling.

Here we demonstrated that individual susceptibility to EIBS could be, at least partially, supported by epigenetic mechanisms. Indeed, we highlighted that resistant mice are tolerant to some transcriptional effect of an ethanol challenge. We also demonstrated specific alteration in striatal HDAC activity and in subsequent H4 acetylation in sensitized but not in resistant mice. This study confirms and extends previous data showing a deep involvement of epigenetic mechanisms in ethanol-related behaviors and provides new insights on neuroadaptations occurring during the expression phase of EIBS and, in extension, on relapse to drug-seeking.

## Materials and Methods

### Animals

Female DBA/2J mice were purchased from Janvier (Le Genest Saint Isle, France), housed 10 per cage and kept in a temperature- (21±0.5°C) and humidity-controlled (55±10%) environment under an established photoperiod (07.00–19.00 hours) with free access to food and tap water. All along the experiments, mice were housed 10 per cage. At the beginning of the experiments, mice were 8–9-week-old. Different subjects were used in two separate sets of experiments in which similar behavioral measure has been performed. The first set has been used for blood ethanol levels and PCR array analyses and the second for enzymes activities and immunohistochemistry assessments. Thus EIBS has been assessed in all tested groups.

The number of animals was kept to a minimum, and all efforts were made to avoid making the animals suffering. Experiments were carried out in strict accordance with both the guidelines for Care and Use of Laboratory Animals (NIH) and the European Community regulations for animal use in research (CEE No 86/609) and were also approved by the local ethics committee.

### Drugs

Ethanol (96%, v/v), obtained from Prolabo (Fontenay-sous-Bois, France), was diluted to 20% (v/v) in saline solution 0.9%. All injections were made intraperitoneally (i.p.) in volumes of 1.25 ml per 100 g body weight.

### EIBS Induction

Behavioral sensitization procedure was similar to that previously described [38]. Locomotor activity was assessed in the LE 8811 IR motor activity monitor (Bioseb, Vitrolles, France). Mice were placed in a 40×40×30 cm open field with opaque acrylic walls, transected with infrared photocell beams 2 cm above the floor at 16 sites along each side. Test chambers were shielded from external noise and illuminated with indirect white light (20 lux). Horizontal locomotion was measured from photocell beam interruptions using ActiTrack software (Bioseb).

On the first day of the experiment (habituation day), all mice were i.p. injected with saline solution (12.5 ml/kg), and immediately placed into the open field (n = 60). Locomotor activity was recorded for the next 5 min as done in other studies [39], [40]. Mice were then divided into several groups that were equated in terms of horizontal locomotion. The next day, the sensitization procedure started. During 10 days, mice received once daily i.p. injections of ethanol (2 g/kg) or saline solution (induction phase). At day 10, we retrospectively split ethanol-treated animals in resistant and sensitized groups based on their sensitization score (D10/D1 ratio) as previously described by Boudreau and Wolf [41]. Ethanol-treated mice were therefore considered as sensitized if their increase in locomotor activity exceeded the coefficient of variance of the control group [41]. About 70% of ethanol-treated mice exhibited EIBS. After the last day of sensitization (D10), mice were let undisturbed in their homecages for 7 days. At day 17 (ethanol challenge), ethanol-treated mice (resistant and sensitized groups; n = 40) received a single i.p. injection of 2 g/kg ethanol. Mice receiving saline solution during the induction phase were split in two groups, *i.e*. control (n = 10) and acute (n = 10) groups, and were i.p. injected with saline or 2 g/kg ethanol, respectively.

### Ethanol Metabolism

At day 17, mice were euthanized 30 min after a 2 g/kg ethanol injection and blood was collected. BECs were measured in plasma with the AM1 Alcohol Analyser (Analox Instruments, IMLAB, Lille, France) (n = 9 per group). The precision of this assay is 1– 2%, sensitivity is 0.1 mg/100 ml, and the curve is linear up to 400 mg/100 ml.

### Real-time PCR Array

Thirty minutes after challenge injection on day 17, striata were removed to perform real-time PCR (n = 4 per group). Total RNAs were then harvested from striatal tissues using the Trizol method (Invitrogen, Carlsbad, CA, USA) (one mouse for one sample). After further purification using the RNeasy Lipid Tissue Mini kit (Qiagen, Valencia, CA, USA), contaminating genomic DNA was eliminated by treatment with DNAse I (Qiagen) and cDNA were synthesized from 1 µg of RNA using RT2 profiler PCR array first strand kit (Qiagen). RT2 Profiler PCR arrays, mouse epigenetic chromatin modification enzymes (PAMM-085-C, SABiosciences) were performed according to the manufacturer’s instructions in the presence of SYBR green master mix (SABiosciences) using the StepOnePlus detection system (Applied Biosystems, Courtabœuf, France). Each array contains a panel of 96 primer sets for a thoroughly researched set of 84 genes, 3 RNA and PCR quality controls and 5 housekeeping genes for variations in amounts of input mRNA (*gusb, hprt1, hsp90ab1, gapdh, actb*). No value was removed from data analysis with PCR array data analysis template downloaded from the Superarray Web site (www.sabiosciences.com) using the 2^−ΔΔCq^ method.

### Epigenetic-related Enzymes Activities

At day 17, mice were challenged with ethanol or saline solution and euthanized 30 min later. Striatum, PFC, amygdala and hippocampus were isolated to extract nuclear and cytosolic proteins using a commercial nuclear extraction kit (Imgenex, San Diego, CA, USA). Toward this end, brain tissues were rinsed with PBS/PMSF 1X and homogenized in hypotonic buffer. After incubation on ice for 30 min and centrifugation (10 min, 4°C, 10,000 rpm), cytosolic fractions were obtained and conserved at 4°C. The pellets were then homogenized in nuclear lysis buffer and incubated 30 min at 4°C. After centrifugation (10 min, 4°C, 14,000 rpm), nuclear fractions were obtained and conserved at −80°C. Nuclear and cytosolic proteins concentrations were quantified using a Bradford assay (Bio-Rad, Hercules, CA, USA).

Global HDAC (n = 4/5 per group) and HAT (n = 4/5 per group) activities were evaluated in nuclear and cytosolic fractions using commercial HAT activity colorimetric and HDAC activity fluorometric assay kits, respectively according to the manufacturer’s instructions (BioVision, Milpitas, CA, USA). Global DNMT activity (n = 5 per group) was evaluated in nuclear fractions using commercial DNMT Activity/Inhibition Assay kit according to the manufacturer’s instruction (Active Motif, La Hulpe, Belgium). HDAC, HAT and DNMT activities were performed in the nuclear fraction due to their role on gene regulation. HDAC and HAT activities were also assessed in cytosolic fractions due to their actions on non-histone targets.

### Immunohistochemistry

At day 17, mice were challenged with ethanol or saline solution and transcardially perfused 30 min later. Brains were removed, frozen and cut in the frontal plane into 50-µm-thick serial sections (n = 4 per group). Free-floating sections were incubated first with fresh 0.3% H_2_O_2_ for 30 min at room temperature and second with primary antibody directed against acetyl-H4 (Lys12, 1∶7500, Millipore, Molsheim, France) or NeuN (specific neuronal nuclei marker; 1∶1000, Millipore) diluted in blocking solution (phosphate buffer saline 0.1 M; 0.1% BSA; 0.2% Triton-X-100; 2% serum) overnight at room temperature on a shaker. NeuN labelling was performed to evaluate ethanol-induced neurotoxicity. Next, a biotin-labelled secondary antibody diluted in blocking solution was added for 2 h at room temperature on a shaker (Vector, Paris, France). Tissues labelling were detected using the avidin biotinylated enzyme complex and diaminobenzidine method (Vector). Slices were mounted and coverslipped before analysis with an optical microscope Leica (DM 4000 M; Germany) coupled to a camera (Infinity 2; BFI OPTiLAS, France) and to an acquisition system Mercator (ExploraNova, France). Immunoreactive neurons were counted bilaterally in the Nac core and shell and in the dorso-lateral and dorso-medial striatum [42] **(Figure S1)**.

### Statistical Analyses

All data analyses were conducted using SigmaStat2.0 software. Data from the sensitization experiments were analysed with two-way repeated measure analysis of variance (RM-ANOVA) followed by Tukey’s *post-hoc* tests. Data from day H, from induction (D1–10) and from expression (D1, D10 and D17) were analyzed separately. Data from BECs, HDAC, HAT and DNMT activities and immunohistochemistry were analysed with 1-way ANOVA followed by a Tukey’s *post-hoc* tests. Data from PCR experiments were analysed with 1-way ANOVA followed by Tukey’s *post-hoc* tests. Since 84 genes were examined, it was necessary to make an adjustment in the level of significance. However a *P<*0.0006 (α/84 analysed genes, Bonferroni correction) is considered too conservative, thus we chose an intermediate level of significance corresponding to α/number of regulated genes (*i.e.* α/40). Regulations that reached the classical level of significance (*P<*0.05) but do not pass the Bonferroni correction were indicated.

## Supporting Information

Figure S1 **Schematic drawings of mouse coronal sections.** The regions of interest for measurement of acetyl-H4K12- and NeuN-positive cells are indicated by hatched areas.
(TIF)

Figure S2 **Expression of EIBS is not due to ethanol metabolism or food intake alteration.** During 10 days, mice received daily i.p. injections of saline or ethanol (2 g/kg) solution. Blood ethanol concentrations (**a**) and mice body weights (**b**) were assessed 30 min after saline or ethanol challenge (2 g/kg) at day 17. Each histogram represent mean (± SEM) of 9–10 animals per group.
(TIF)

Figure S3 **Expression of EIBS is not associated with ethanol-induced neurotoxicity.** During 10 days, mice received daily i.p. injections of saline or ethanol (2 g/kg) solution. At day 17, mice were challenged with saline or ethanol (2 g/kg) solution and transcardially perfused 30 min later to perform immunohistochemistry (n = 4 per group). (**a**) Photomicrographs illustrating the immunolabelling for NeuN in the core of the Nac. The number of NeuN positive cells are represented as mean values (± SEM) in the core (**b**) and in the shell (**c**) of the Nac or in the dorso-lateral (**d**) and dorso-medial (**e**) striatum. Nac, nucleus accumbens.
(TIF)

Table S1 **Functional classification of striatal epigenetic-related genes studied in acute, resistant and sensitized mice during EIBS expression phase.** During 10 days, mice received daily i.p. injections of saline or ethanol (2 g/kg) solution. At day 17, 30 min after saline or ethanol challenge (2 g/kg), striata were isolated to perform real-time PCR (n = 4 per group). Means (± SEM) of regulated genes expression in striatum are expressed as percentages of control *P<0.05, **P<0.01, ***P<0.001 *vs* control; ^#^ *P<*0.05, ^##^ *P<*0.01, ^###^ *P<*0.001 *vs* acute; ^†^ *P<*0.05, ^††^ *P<*0.01, ^†††^ *P<*0.001 *vs* resistant groups. Regulations that pass the Bonferroni correction are indicated in grey.
(XLS)

## References

[1] Robinson TE, Berridge KC (1993) The neural basis of drug craving: an incentive-sensitization theory of addiction. Brain Res Brain Res Rev 18: 247–291. 8401595 10.1016/0165-0173(93)90013-p

[2] Steketee JD, Kalivas PW (2011) Drug wanting: behavioral sensitization and relapse to drug-seeking behavior. Pharmacol Rev 63: 348–365. 21490129 10.1124/pr.109.001933 PMC3082449

[3] Vanderschuren LJ, Pierce RC (2010) Sensitization processes in drug addiction. Curr Top Behav Neurosci 3: 179–195. 21161753 10.1007/7854_2009_21

[4] Cador M, Bjijou Y, Stinus L (1995) Evidence of a complete independence of the neurobiological substrates for the induction and expression of behavioral sensitization to amphetamine. Neuroscience 65: 385–395. 7777156 10.1016/0306-4522(94)00524-9

[5] Perugini M, Vezina P (1994) Amphetamine administered to the ventral tegmental area sensitizes rats to the locomotor effects of nucleus accumbens amphetamine. J Pharmacol Exp Ther 270: 690–696. 8071860

[6] Camarini R, Nogueira Pires ML, Calil HM (2000) Involvement of the opioid system in the development and expression of sensitization to the locomotor-activating effect of ethanol. Int J Neuropsychopharmacol 3: 303–309. 11343608 10.1017/S146114570000211X

[7] Harrison SJ, Nobrega JN (2009) A functional role for the dopamine D3 receptor in the induction and expression of behavioural sensitization to ethanol in mice. Psychopharmacology (Berl) 207: 47–56. 19662386 10.1007/s00213-009-1629-x

[8] Kim AK, Souza-Formigoni ML (2010) Disulfiram impairs the development of behavioural sensitization to the stimulant effect of ethanol. Behav Brain Res 207: 441–446. 19891992 10.1016/j.bbr.2009.10.032

[9] Fee JR, Sparta DR, Picker MJ, Thiele TE (2007) Corticotropin releasing factor-1 receptor antagonist, CP-154,526, blocks the expression of ethanol-induced behavioral sensitization in DBA/2J mice. Neuroscience 150: 14–21. 17919825 10.1016/j.neuroscience.2007.08.027 PMC2194653

[10] Abrahao KP, Quadros IM, Souza-Formigoni ML (2011) Nucleus accumbens dopamine D(1) receptors regulate the expression of ethanol-induced behavioural sensitization. Int J Neuropsychopharmacol 14: 175–185. 20426882 10.1017/S1461145710000441

[11] Masur J, dos Santos HM (1988) Response variability of ethanol-induced locomotor activation in mice. Psychopharmacology (Berl) 96: 547–550. 3149779 10.1007/BF02180038

[12] Quadros IM, Hipolide DC, Frussa-Filho R, De Lucca EM, Nobrega JN, et al (2002) Resistance to ethanol sensitization is associated with increased NMDA receptor binding in specific brain areas. Eur J Pharmacol 442: 55–61. 12020682 10.1016/s0014-2999(02)01503-0

[13] Quadros IM, Nobrega JN, Hipolide DC, de Lucca EM, Souza-Formigoni ML (2002) Differential propensity to ethanol sensitization is not associated with altered binding to D1 receptors or dopamine transporters in mouse brain. Addict Biol 7: 291–299. 12126488 10.1080/13556210220139505

[14] Souza-Formigoni ML, De Lucca EM, Hipolide DC, Enns SC, Oliveira MG, et al (1999) Sensitization to ethanol’s stimulant effect is associated with region-specific increases in brain D2 receptor binding. Psychopharmacology (Berl) 146: 262–267. 10541725 10.1007/s002130051115

[15] Pandey SC, Ugale R, Zhang H, Tang L, Prakash A (2008) Brain chromatin remodeling: a novel mechanism of alcoholism. J Neurosci 28: 3729–3737. 18385331 10.1523/JNEUROSCI.5731-07.2008 PMC6671100

[16] Sakharkar AJ, Zhang H, Tang L, Shi G, Pandey SC (2012) Histone deacetylases (HDAC)-induced histone modifications in the amygdala: a role in rapid tolerance to the anxiolytic effects of ethanol. Alcohol Clin Exp Res 36: 61–71. 21790673 10.1111/j.1530-0277.2011.01581.x PMC3208078

[17] Pascual M, Do Couto BR, Alfonso-Loeches S, Aguilar MA, Rodriguez-Arias M, et al (2012) Changes in histone acetylation in the prefrontal cortex of ethanol-exposed adolescent rats are associated with ethanol-induced place conditioning. Neuropharmacology 62: 2308–2318. 10.1016/j.neuropharm.2012.01.011 22349397

[18] Sanchis-Segura C, Lopez-Atalaya JP, Barco A (2009) Selective boosting of transcriptional and behavioral responses to drugs of abuse by histone deacetylase inhibition. Neuropsychopharmacology 34: 2642–2654. 19727068 10.1038/npp.2009.125

[19] Fuchs RA, Evans KA, Ledford CC, Parker MP, Case JM, et al (2005) The role of the dorsomedial prefrontal cortex, basolateral amygdala, and dorsal hippocampus in contextual reinstatement of cocaine seeking in rats. Neuropsychopharmacology 30: 296–309. 15483559 10.1038/sj.npp.1300579

[20] Svedruzic ZM (2011) Dnmt1 structure and function. Prog Mol Biol Transl Sci 101: 221–254. 21507353 10.1016/B978-0-12-387685-0.00006-8

[21] Anier K, Malinovskaja K, Aonurm-Helm A, Zharkovsky A, Kalda A (2010) DNA methylation regulates cocaine-induced behavioral sensitization in mice. Neuropsychopharmacology 35: 2450–2461. 20720536 10.1038/npp.2010.128 PMC3055323

[22] Tian W, Zhao M, Li M, Song T, Zhang M, et al (2012) Reversal of cocaine-conditioned place preference through methyl supplementation in mice: altering global DNA methylation in the prefrontal cortex. PLoS One 7: e33435. 22438930 10.1371/journal.pone.0033435 PMC3306398

[23] Kim BJ, Kang KM, Jung SY, Choi HK, Seo JH, et al (2008) Esco2 is a novel corepressor that associates with various chromatin modifying enzymes. Biochem Biophys Res Commun 372: 298–304. 18501190 10.1016/j.bbrc.2008.05.056

[24] Brami-Cherrier K, Roze E, Girault JA, Betuing S, Caboche J (2009) Role of the ERK/MSK1 signalling pathway in chromatin remodelling and brain responses to drugs of abuse. J Neurochem 108: 1323–1335. 19183268 10.1111/j.1471-4159.2009.05879.x

[25] Gutierrez-Mecinas M, Trollope AF, Collins A, Morfett H, Hesketh SA, et al (2011) Long-lasting behavioral responses to stress involve a direct interaction of glucocorticoid receptors with ERK1/2-MSK1-Elk-1 signaling. Proc Natl Acad Sci U S A 108: 13806–13811. 21808001 10.1073/pnas.1104383108 PMC3158237

[26] DiRocco DP, Scheiner ZS, Sindreu CB, Chan GC, Storm DR (2009) A role for calmodulin-stimulated adenylyl cyclases in cocaine sensitization. J Neurosci 29: 2393–2403. 19244515 10.1523/JNEUROSCI.4356-08.2009 PMC2678191

[27] Host L, Dietrich JB, Carouge D, Aunis D, Zwiller J (2011) Cocaine self-administration alters the expression of chromatin-remodelling proteins; modulation by histone deacetylase inhibition. J Psychopharmacol 25: 222–229. 19939859 10.1177/0269881109348173

[28] Martin TA, Jayanthi S, McCoy MT, Brannock C, Ladenheim B, et al (2012) Methamphetamine causes differential alterations in gene expression and patterns of histone acetylation/hypoacetylation in the rat nucleus accumbens. PLoS One 7: e34236. 22470541 10.1371/journal.pone.0034236 PMC3314616

[29] de Ruijter AJ, van Gennip AH, Caron HN, Kemp S, van Kuilenburg AB (2003) Histone deacetylases (HDACs): characterization of the classical HDAC family. Biochem J 370: 737–749. 12429021 10.1042/BJ20021321 PMC1223209

[30] Pascual M, Boix J, Felipo V, Guerri C (2009) Repeated alcohol administration during adolescence causes changes in the mesolimbic dopaminergic and glutamatergic systems and promotes alcohol intake in the adult rat. J Neurochem 108: 920–931. 19077056 10.1111/j.1471-4159.2008.05835.x

[31] Kazantsev AG, Thompson LM (2008) Therapeutic application of histone deacetylase inhibitors for central nervous system disorders. Nat Rev Drug Discov 7: 854–868. 18827828 10.1038/nrd2681

[32] Shukla SD, Velazquez J, French SW, Lu SC, Ticku MK, et al (2008) Emerging role of epigenetics in the actions of alcohol. Alcohol Clin Exp Res 32: 1525–1534. 18616668 10.1111/j.1530-0277.2008.00729.x

[33] Kalda A, Heidmets LT, Shen HY, Zharkovsky A, Chen JF (2007) Histone deacetylase inhibitors modulates the induction and expression of amphetamine-induced behavioral sensitization partially through an associated learning of the environment in mice. Behav Brain Res 181: 76–84. 17477979 10.1016/j.bbr.2007.03.027 PMC2992845

[34] Levine AA, Guan Z, Barco A, Xu S, Kandel ER, et al (2005) CREB-binding protein controls response to cocaine by acetylating histones at the fosB promoter in the mouse striatum. Proc Natl Acad Sci U S A 102: 19186–19191. 16380431 10.1073/pnas.0509735102 PMC1323217

[35] Cadoni C, Di Chiara G (2000) Differential changes in accumbens shell and core dopamine in behavioral sensitization to nicotine. Eur J Pharmacol 387: R23–25. 10650185 10.1016/s0014-2999(99)00843-2

[36] Nordquist RE, Voorn P, de Mooij-van Malsen JG, Joosten RN, Pennartz CM, et al (2007) Augmented reinforcer value and accelerated habit formation after repeated amphetamine treatment. Eur Neuropsychopharmacol 17: 532–540. 17275266 10.1016/j.euroneuro.2006.12.005

[37] Xu CM, Wang J, Wu P, Xue YX, Zhu WL, et al (2011) Glycogen synthase kinase 3beta in the nucleus accumbens core is critical for methamphetamine-induced behavioral sensitization. J Neurochem 118: 126–139. 21517846 10.1111/j.1471-4159.2011.07281.x

[38] Simon O’Brien E, Legastelois R, Houchi H, Vilpoux C, Alaux-Cantin S, et al (2011) Fluoxetine, desipramine, and the dual antidepressant milnacipran reduce alcohol self-administration and/or relapse in dependent rats. Neuropsychopharmacology 36: 1518–1530. 21430652 10.1038/npp.2011.37 PMC3096819

[39] de Araujo NP, Fukushiro DF, Grassl C, Hipolide DC, Souza-Formigoni ML, et al (2009) Ethanol-induced behavioral sensitization is associated with dopamine receptor changes in the mouse olfactory tubercle. Physiol Behav 96: 12–17. 18761028 10.1016/j.physbeh.2008.07.029

[40] Didone V, Quoilin C, Tirelli E, Quertemont E (2008) Parametric analysis of the development and expression of ethanol-induced behavioral sensitization in female Swiss mice: effects of dose, injection schedule, and test context. Psychopharmacology (Berl) 201: 249–260. 18685830 10.1007/s00213-008-1266-9

[41] Boudreau AC, Wolf ME (2005) Behavioral sensitization to cocaine is associated with increased AMPA receptor surface expression in the nucleus accumbens. J Neurosci 25: 9144–9151. 16207873 10.1523/JNEUROSCI.2252-05.2005 PMC6725751

[42] Paxinos G, Watson C (1998) The rat brain in stereotaxic coordinates. 4^th^ Ed. Academic Press, San Diego, CA.

