# Supplementary figures and images for: Expression of Ethanol-Induced Behavioral Sensitization Is Associated with Alteration of Chromatin Remodeling in Mice
Source: PLoS One. 2012 Oct 22;7(10):e47527. doi: 10.1371/journal.pone.0047527 (PMC3478273; doi:10.1371/journal.pone.0047527)

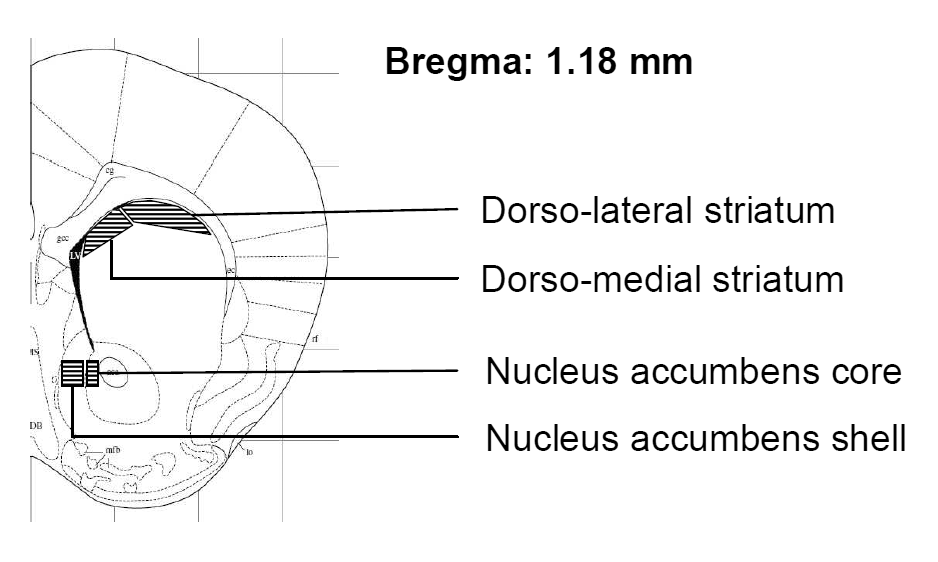

Supplement: Figure S1 — Schematic drawings of mouse coronal sections. The regions of interest for measurement of acetyl-H4K12- and NeuN-positive cells are indicated by hatched areas. (TIF) [file pone.0047527.s001.tif]

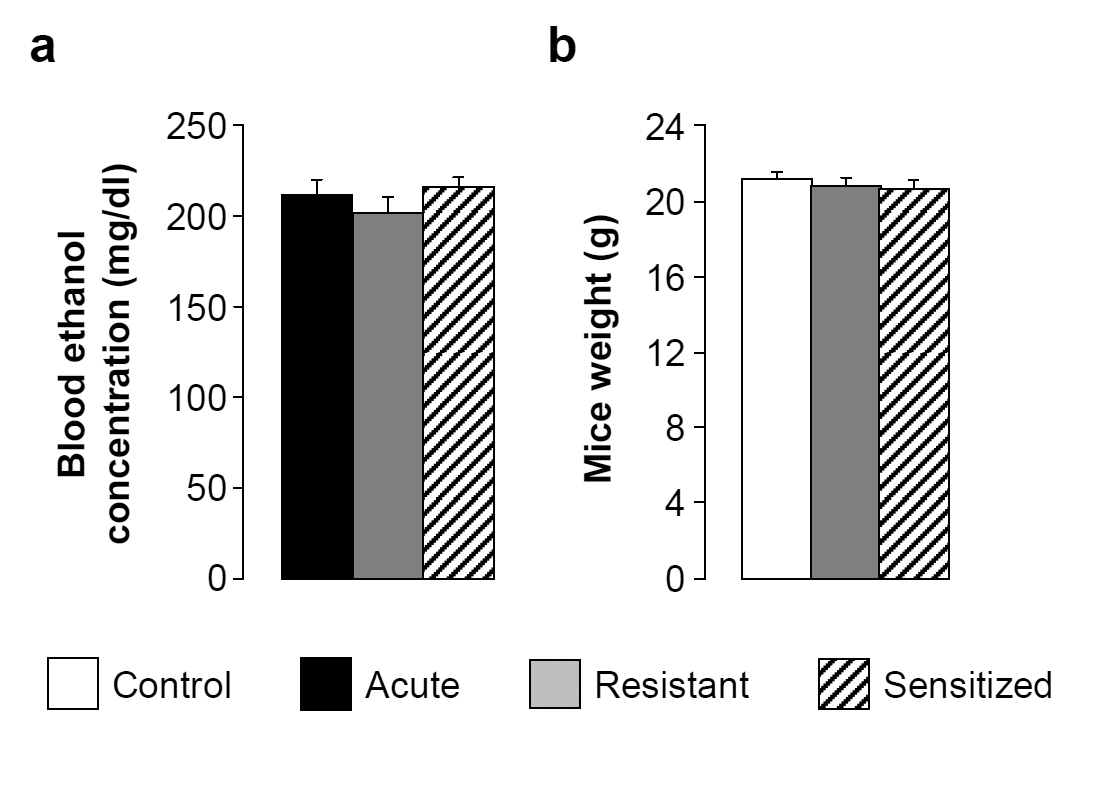

Supplement: Figure S2 — Expression of EIBS is not due to ethanol metabolism or food intake alteration. During 10 days, mice received daily i.p. injections of saline or ethanol (2 g/kg) solution. Blood ethanol concentrations (a) and mice body weights (b) were assessed 30 min after saline or ethanol challenge (2 g/kg) at day 17. Each histogram represent mean (± SEM) of 9–10 animals per group. (TIF) [file pone.0047527.s002.tif]

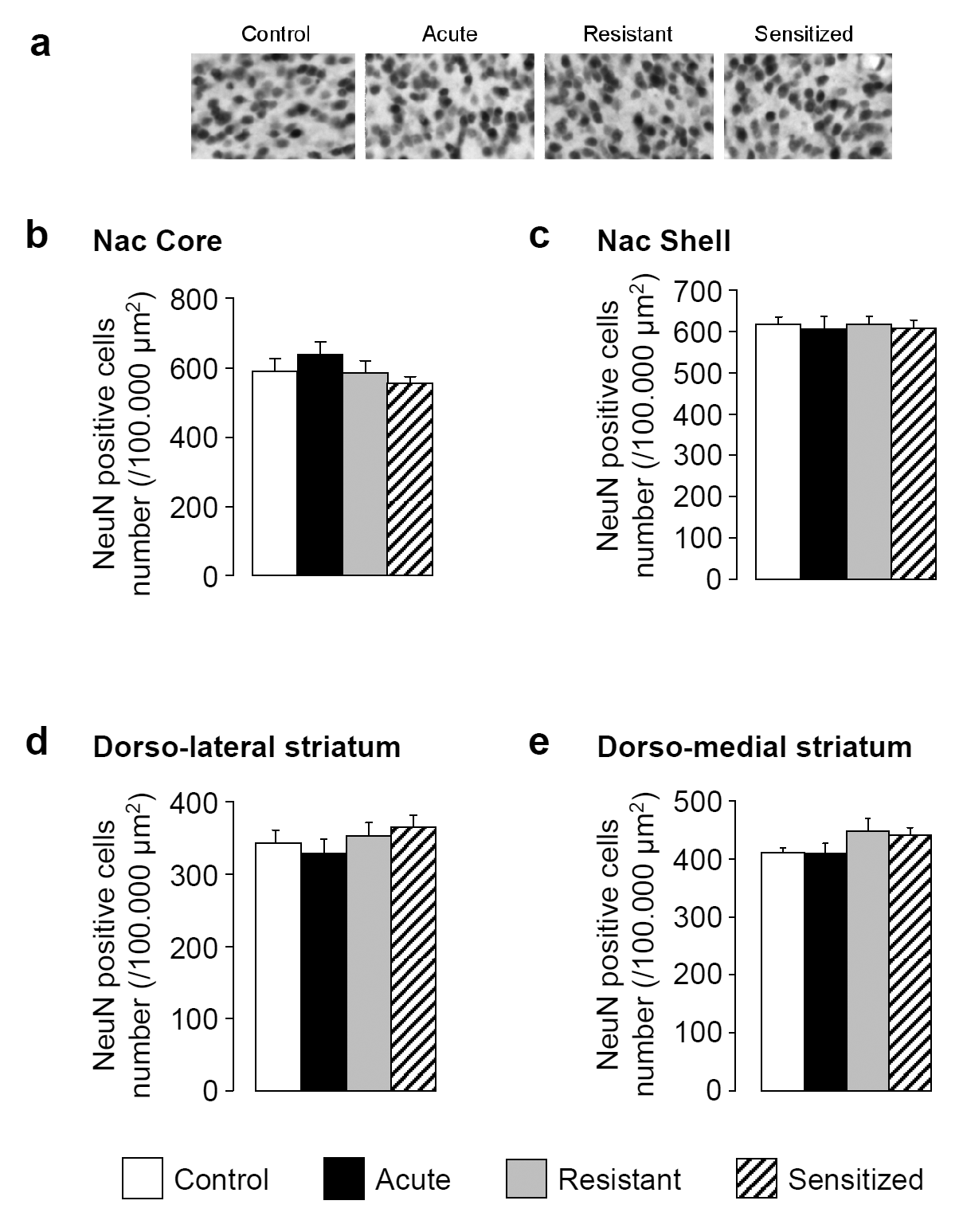

Supplement: Figure S3 — Expression of EIBS is not associated with ethanol-induced neurotoxicity. During 10 days, mice received daily i.p. injections of saline or ethanol (2 g/kg) solution. At day 17, mice were challenged with saline or ethanol (2 g/kg) solution and transcardially perfused 30 min later to perform immunohistochemistry (n = 4 per group). (a) Photomicrographs illustrating the immunolabelling for NeuN in the core of the Nac. The number of NeuN positive cells are represented as mean values (± SEM) in the core (b) and in the shell (c) of the Nac or in the dorso-lateral (d) and dorso-medial (e) striatum. Nac, nucleus accumbens. (TIF) [file pone.0047527.s003.tif]
